# Dental Implants with External Hex Inclined Shoulder in Full-Arch Immediate Loading Rehabilitations of the Maxilla

**DOI:** 10.3390/dj12050131

**Published:** 2024-05-08

**Authors:** Francesco Bagnasco, Paolo Pesce, Domenico Baldi, Francesco Motta, Francesco Pera, Nicola De Angelis, Maria Menini

**Affiliations:** 1 Department of Surgical Sciences (DISC), Dental School, University of Genoa, 16126 Genova, Italy; fcbagna5@hotmail.it (F.B.); paolo.pesce@unige.it (P.P.); baldi.domenico@unige.it (D.B.); francesco.pera1@gmail.com (F.P.); maria.menini@unige.it (M.M.); 2 Private Practice, Biella 13900, Italy; motta.gol@gmail.com

**Keywords:** coaxial implants, tilted implants, full-arch, immediate dental implant loading, case report

## Abstract

*Background:* Coaxial implants with an inclined neck might overcome some problems related to angulation of the implant axis when using tilted implants. Therefore, the aim of the present work was to conduct a narrative review of the current literature and to present a case series comparing traditional and coaxial external hex implants in full-arch immediate loading rehabilitations of the maxilla. *Methods:* A total of 13 external hex tapered implants (Southern Implants) was inserted in the upper jaw of 3 patients. Each patient received two tilted implants in distal sites. In one randomly selected quadrant, the tilted implant was a standard implant, while a Co-Axis^®^ implant with a 24° inclination of the implant shoulder was inserted on the other hemi-arch. Straight conical abutments were screwed on coaxial implants while multiunit abutments of appropriate inclination were screwed as needed on the other implants to correct their axes. Peri-implant bone level was recorded radiographically at T0 (delivery of the immediate loading prosthesis), and at 3, 6, 12, and 24 months of healing and then annually. Plaque index, probing depth, and bleeding on probing were also evaluated. Cumulative implant survival rate (CSR) was calculated, and biological or technical complications were recorded as well as the operator satisfaction towards the use of coaxial implants. *Results*: The preliminary data collected did not show significant differences in peri-implant tissues health and maintenance over time between the two implant types. No implants failed, and both implant types proved to be favorable for full-arch rehabilitation using tilted implants. Coaxial implants facilitated the prosthodontic procedures. However, a learning curve is required in order to optimize their insertion. *Conclusions:* Both implants proved to be reliable and suitable for achieving clinical success in full-arch immediate loading rehabilitations, but further research with longer follow-up and larger sample size is needed to confirm these preliminary outcomes.

## 1. Introduction

Immediate loading full-arch rehabilitations have become increasingly prevalent procedures since the 2000s [1,2]. Over time, this method has demonstrated a high level of success predictability in both the medium and long term [2].

By strategically placing a small number of implants to achieve proper load distribution, phonetic and aesthetic chewing abilities can be restored within just 24 to 48 h [2]. For patients who are completely edentulous or have severely compromised residual dentition, alveolar bone resorption can pose a challenge for implant-supported rehabilitation, especially in areas near the mandibular nerve and maxillary sinus. Traditionally, regenerative procedures such as guided bone regeneration (GBR) or sinus lifts have been performed to increase local bone availability [3]. However, these procedures result in increased surgical sessions, morbidity, intraoperative complications, and longer rehabilitation timelines [4].

To overcome the need for invasive regenerative procedures, short implants have been proposed for single and partial rehabilitations [5,6]. Conversely, tilted implants have been introduced for full-arch rehabilitation, allowing the avoidance of critical anatomical structures such as the maxillary sinus and mandibular nerve in atrophic jaws [7,8,9].

The use of tilted implants to support immediately loaded fixed prostheses for edentulous upper jaw rehabilitation is now regarded as a reliable technique, demonstrating comparable failure rates and bone resorption levels to vertical implants [10].

Initially, tilted implants were implemented to decrease the distal prosthetic cantilever, thereby influencing the forces transferred to the abutments of implant-supported fixed prostheses and the surrounding bone. Placement of tilted implants in the distal sectors of the upper jaw facilitates the use of longer implants, thereby enhancing primary stability and distributing loads more effectively in regions subjected to substantial masticatory forces.

To correct the angulation of tilted implants, multiunit abutments (MUAs) have been developed to achieve perfect parallelism between all supporting implants. The use of MUAs allows the interface of the implant–prosthetic connection to be at the tissue level, reducing the possibility of bacterial colonization, the resulting inflammatory response, and possible peri-implant complications [11]. While MUAs can improve the inclination of implant emergence, they may not always ensure perfect parallelism, leading to increased forces around the head of inclined implants and a possible increase in marginal bone resorption [12].

A systematic review by Omori et al. in 2020 [13], including nine nonrandomized cohort studies (total: 797 patients who received 4127 implants), found similar mechanical complications between angulated and straight abutments, with the most common failures being screw loosening and screw fractures. In contrast, a significantly higher failure rate and greater mean bone loss were reported for implants supporting angulated abutments compared to straight abutments at 1 year after insertion [13].

This contrasts with the previously mentioned systematic reviews on biological and technical complications of tilted compared to straight implants [7].

Omori et al. hypothesized that their outcome might be due to eccentric loading of the implants with angulated abutments and the subsequent distribution of stresses or to microbiological factors pertaining to the peri-implant area [13].

These loading forces, which may induce flexion or micromovement of the implant–abutment system, may be associated with increased micro-gap and a “pump effect” between the implant interior and peri-implant tissues [14].

It must also be considered that the use of additional components (MUAs) not only increases the costs of rehabilitation but also complicates clinical procedures. In particular, selecting the MUAs of the best angulation and placing them in the proper position immediately after surgery might be challenging due to bleeding and subcrestal positioning of the implant head, and might lead to additional patient discomfort after surgery. Additionally, even if dependent on the implant angulation and on the type (model, shape, height) of MUA, the greater the MUA’s inclination, the higher their prosthetic neck, which, in the presence of significant bone and gingival resorption, may lead to exposure of the metal component and potential esthetic failure [15].

To address these issues, implants with a preinclined head have been developed [16,17,18]. They could eliminate the need for MUAs to correct implant inclination when using implants inclined at 12° or 24°. This might simplify prosthodontic procedures and allow screw-retained restorations.

A narrative review of the literature searching PubMed, EMBASE, and Google Scholar was conducted of the available clinical studies related to the topic, using the following keywords in different combinations: “dental implants”, “coaxial implant”, “inclined/angled neck”, “inclined platform”, and “full-arch rehabilitations”. Only eight clinical studies were found [18,19,20,21,22,23,24,25], and the majority of them [19,22,23,24,25] investigated single-implant rehabilitations.

All the studies investigating single implants were prospective clinical studies except for one, which was a case series [25]. One study [21] was a case report on partial rehabilitation, and only one study was a split-mouth study on full-arch immediate loading rehabilitations [18]. All the studies were prospective and showed good clinical outcomes for coaxial implants.

Van Weehaeghe et al. [20] compared conventional implants with angulated abutments to tilted implants with an angulated connection. Twenty patients received treatment in the edentulous mandible, with various implant configurations. After a 48-month follow-up, results showed no significant differences in implant survival, marginal bone loss (MBL), periodontal indices, patient satisfaction, or complications between implants restored on abutment or implant level, or between different configurations of angulated implants. Posterior implants had less MBL compared to anterior implants. There was no significant difference in MBL between implants restored with zirconia or porcelain-fused-to-metal (PFM) bridges. Zirconia bridges showed less plaque accumulation compared to PFM bridges. Overall, patients were highly satisfied. The study suggests that implants with angulated connections may offer stronger connections without affecting MBL and that zirconia may reduce plaque accumulation.

Brown et al. [19] aimed to assess the effectiveness of immediately placing and restoring a new implant with a 12°-angled prosthodontic platform in extraction sockets of the aesthetic zone of the upper jaw. Tapered, roughened surface implants of 4 mm or 5 mm diameter were placed in 27 participants requiring replacement of single anterior maxillary teeth. Provisional crowns were placed within 4 h, followed by definitive crowns at 8 weeks. Results showed successful outcomes with bone gain, stable mucosal margins, and favorable aesthetics. The novel implant design facilitated prosthodontic maintenance, and the implant crowns exhibited high success rates with minimal issues over one year.

Vandeweghe et al. [20] evaluated the same implant of Brown et al., focusing on bone loss, peri-implant health, and aesthetic outcomes after 1 year. Fifteen implants in 14 patients were immediately loaded with screw-retained full ceramic crowns. Results showed that all implants survived, with mean bone loss of 1.20 mm and stable plaque levels. Bleeding levels decreased initially but remained constant afterward. Four cases of screw loosening and one crown chipping occurred. Patients reported improved wellbeing. Midfacial recession averaged 0.37 mm, with slight changes in papilla fill. Overall, the coaxial implant exhibited good clinical outcomes with stable bone levels and satisfactory aesthetics over 1 year, despite some minor issues with screw loosening and crown chipping

Ma et al. [22] investigated the 5-year clinical success of using tilted implants immediately after tooth extraction, followed by all-ceramic crowns for tooth replacement. Twenty-seven participants received 28 single implant crowns in the maxillary aesthetic zone. Immediate implant placement with titanium implants featuring a 12°-angled platform was followed by provisional crowns within 4 h and definitive crowns at 8 weeks. Data collected over 5 years showed minimal changes in marginal bone levels and mid-buccal mucosal levels, with increased implant stability quotient values over time. Prosthodontic maintenance issues were mostly observed in the first year. The study suggests that using tilted implants with zirconia abutments can be a successful rehabilitation option for single missing teeth in the anterior maxilla.

Chu analyzed the tilted platform in two different works. One prospective cohort [23] clinical study evaluated a macro hybrid implant designed for immediate tooth replacement in maxillary anterior post-extraction sockets. Thirty-three patients received these implants, which combine cylindrical and tapered shapes with a subcrestal angle correction feature. Results showed high implant survival rates, stable labial bone plate thickness, preserved interproximal bone crest thickness, and excellent pink esthetic scores. The implant achieved mean insertion torque values conducive to stability, and no failures occurred during the 1-year follow-up. The study suggests that this hybrid implant design is beneficial for achieving successful implant survival and esthetic outcomes in immediate tooth replacement therapy, particularly in preserving labial plate and papilla without tissue loss or discoloration. Another prospective study [24] compared tilted platform implants to conventional platform-switch-design implants in maxillary anterior post-extraction sockets. Results from 29 patients showed that tilted platform implants led to a greater increase in buccal soft tissue thickness compared to conventional implants, independent of periodontal phenotype.

In a recently published study using three-dimensional finite element analysis (3D-FEA), Aktas and Diker [16] delved into the biomechanical efficacy of an inclined implant shoulder design within the context of all-on-four treatment. Their findings indicate superior biomechanical performance across various clinical structures examined, including peri-implant bone, prosthodontic components, and implants, with the exception of posterior abutment bodies, which exhibited comparable behavior [16]. The study highlights that the inclined shoulder design necessitates positioning the distal portion of the implant shoulder below the alveolar bone level or alternatively placing the mesial portion of the implant shoulder above the bone crest in cases of tilted implants with a standard shoulder design. Conversely, an inclined shoulder design results in increased contact surface area between the implant shoulder and marginal bone [16].

The use of coaxial implants with an inclined neck might reduce costs for clinicians and avoid the potential problems mentioned above. A previous prospective, split-mouth study on 20 patients demonstrated optimal outcomes for coaxial implants used for all-on-four rehabilitation of the lower jaw [18]. However, no significant differences in implant survival, marginal bone loss, periodontal indices, patients’ satisfaction, or complications were found between conventional tilted implants with angulated abutments and angulated implants without abutments.

To the authors’ knowledge, no studies have been published yet on the use of coaxial implants in full-arch immediate loading rehabilitations of the upper jaw. The aim of the present case series is to compare the use of tilted implants with a standard shoulder design and tilted coaxial implants with an inclined shoulder in full-arch immediate loading rehabilitations of the maxilla.

## 2. Materials and Methods

The study was conducted in accordance with the Declaration of Helsinki, and approved by the Regional Ethics Committee CER (326/2019 3/8/2020) for studies involving humans.

Between January 2020 and April 2021, in the Division of Implant and Prosthetic Dentistry (Department of Surgical Sciences, DISC) of the University of Genoa, Italy, three patients were consecutively selected for the present research based on the following inclusion criteria: general good medical conditions, without contraindications to oral surgery, significantly unfavorable prognoses for their residual maxillary dentitions, and a desire to be treated with full-arch immediately loaded rehabilitation. Patients were recruited if the treatment planning required the insertion of distal tilted implants at both right and left side of the maxilla.

Each patient provided written informed consent, and three experienced surgeons performed the interventions. Before the day of surgery, patients underwent scaling, root planning, or any necessary periodontal treatment to create an oral environment more favorable to wound healing.

In brief, patients with terminal dentitions required a fixed rehabilitation of their upper jaw in order to restore esthetics and function. They were rehabilitated with fixed full-arch rehabilitations supported by 4–5 immediately loaded implants following the Columbus Bridge Protocol with distal tilted implants [2].

Prior to surgery, a clinical and radiographic study was conducted using orthopantomography (OPT) and cone beam computed tomography (CBCT) to assess bone levels and relationships with anatomical structures. Preoperative antibiotic coverage with amoxicillin 875 mg + clavulanic acid 125 mg, every 8 h for the next 7 days, was prescribed. Implant placement was performed under local anesthesia (4% articaine with 1:100,000 Adrenaline; Alfacaine SP; Dentsply Italy, Rome, Italy).

All the patients recruited presented residual severely compromised teeth and were made edentulous the day of surgery. Remaining and hopeless teeth were extracted, and the alveolar sockets were meticulously debrided. Bone ridges were then regularized, reshaped, and flattened before implant placement using a Lindeman drill.

For implant site preparation, a pilot drill with a diameter of 2 mm was initially used, followed by a sequence of drills provided by the implant manufacturer (Southern Implants, Irene, South Africa). The site under preparation was adjusted depending on bone quality to achieve sufficient torque insertion for optimal primary mechanical stability. No surgical guides were used, and a free hand surgery was performed.

**Figure 1:**
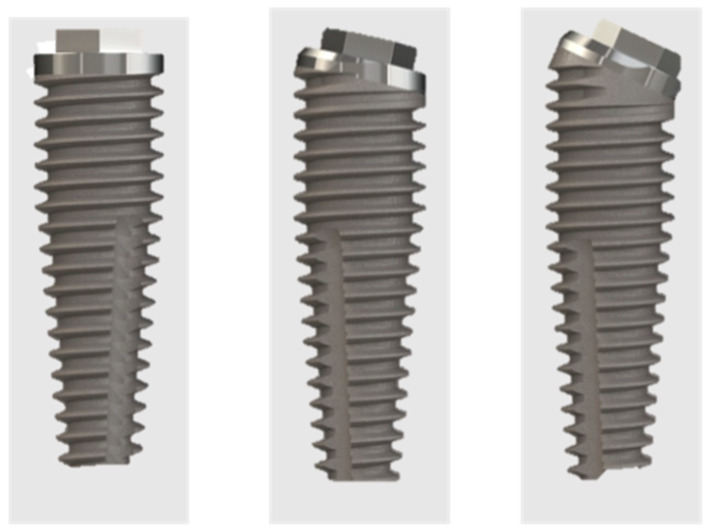
IBT External Hex implant (**left**) and IBR Co-Axis^®^ Implants (Southern Implants) with a 12° (**center**) and 24° (**right**) angulation of the implant shoulder.

In each patient, two different types of external hex tapered implants (IBT External Hex and IBR Co-Axis^®^ Implants, Southern Implants) with identical macro- and micro-topography were placed that differed only in the shoulder design (Figure 1). One of the distal implant sites, where a tilted implant positioning was planned, was randomly selected by extraction and received a Co-Axis^®^ external hexagon implant with a 24° implant platform angulation, while the remaining implant sites received standard external hexagon implants.

All implants were made from grade IV commercially pure titanium and had a diameter of 4.00 mm and a length of 13, 15, or 18 mm, with the only difference being the implant platform angulation at 12° or 24° to the longitudinal axis of the implant.

The implants had a moderately rough surface (Sa 1–2 μm) called Sinergy (Southern Implants), obtained via sandblasting with alumina particles.

Following surgery, conical multiunit abutments (0°, 17°, 30° Abutments, Southern Implants) were immediately screwed to all the implants to correct the inclination of their axis when needed. Straight conical abutments were screwed on coaxial implants, while the tilted implant on the opposite side of the maxilla received an angulated abutment.

A plaster pick-up impression (Snow White plaster, Kerr) was made and PGA 5-0 was used for suturing. Definitive fixed screw-retained prostheses with a metal framework and a composite resin veneering material were delivered within 48 h. All the prostheses had a “natural bridge” configuration, without the need for pink resin simulating soft tissue. All the prostheses had the same occlusal scheme and a careful occlusal check was performed to guarantee an even load distribution. The antagonist arch presented natural teeth (1) or natural teeth and a removable partial denture (2). Two months after surgery, a relining of the prosthesis was conducted if needed.

All surgical and prosthodontic procedures were carried out by expert clinicians of the Prosthodontic and Implant Prosthodontic Division of Genoa University. In particular, the same clinician performing surgical phases also placed multiunit abutments and took the immediate impression. The clinicians were experts in immediate loading rehabilitations using tilted implants but had never placed coaxial implants before.

The patients were Instructed on the appropriate hygienic and dietetic guidelines to be followed during the healing period.

Follow-up visits for check-up and suture removal were arranged for 7–10 days post-surgery. Subsequently, patients were recalled at 14 days, 1 month, 3 months, 6 months, 9 months, 12 months, and then annually for further assessments.

### Outcomes

The primary outcome assessed was the cumulative implant survival rate (CSR). Secondary outcome measures included peri-implant marginal bone loss (MBL), which was evaluated at 0, 3, 6, 12, and 24 months post-loading, as well as assessment of plaque index (PI), probing pocket depth (PPD), and bleeding on probing (BoP) at 3, 6, 12, and 24 months post-loading.

Peri-implant MBL was assessed through intraoral digital periapical radiographs taken with the parallel technique. Measurements were conducted using the implant head as a reference point, both mesially and distally at each implant, using digital software (SUITE V4, OrisWin DG, FONA, Assago, Italy).

Periodontal indexes (PI, PPD, and BoP) were evaluated at four points for each implant using a periodontal UNC 15 probe (Hu-Friedy, Chicago, IL, USA). Prostheses were unscrewed to record these indexes.

BoP was determined by the presence of bleeding (yes/no), while PI was defined as the presence of plaque (yes/no).

In addition, a 6-question multiple choice questionnaire was submitted to the surgeons immediately after taking the immediate loading impression to evaluate their satisfaction towards the use of the two implant types. The following questions were asked:

1. Did you encounter any difficulties during the insertion of the standard implant?
2. Did you encounter any difficulties during the insertion of the coaxial implant?
3. Did you encounter more difficulty during the insertion of the standard or of the coaxial implant?
4. With which implant design did you have more difficulty during the multiunit abutment connection?
5. Do you think that the coaxial implant facilitated the optimization of the prosthodontic axes?
6. During future interventions requiring the insertion of tilted implants, which implant type would you prefer?

In the final section of the questionnaire, the clinicians had the possibility to write free comments.

## 3. Results

**Figure 2:**
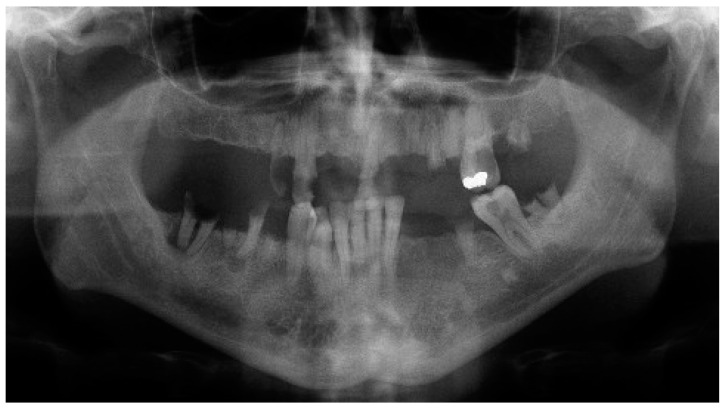
Preoperative panoramic radiograph of patient 1 included in the present research.

**Figure 3:**
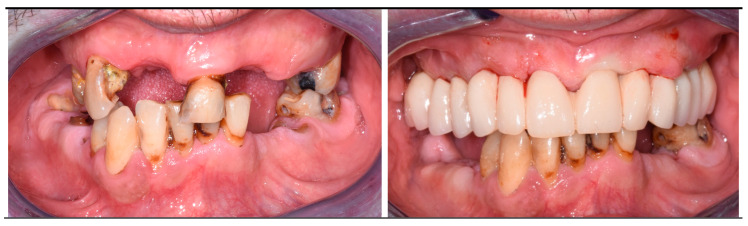
Intraoral pictures taken before treatment (**left**) and at the delivery of the immediate loading prosthesis (**right**) of the same patient of Figure 1.

**Figure 4:**
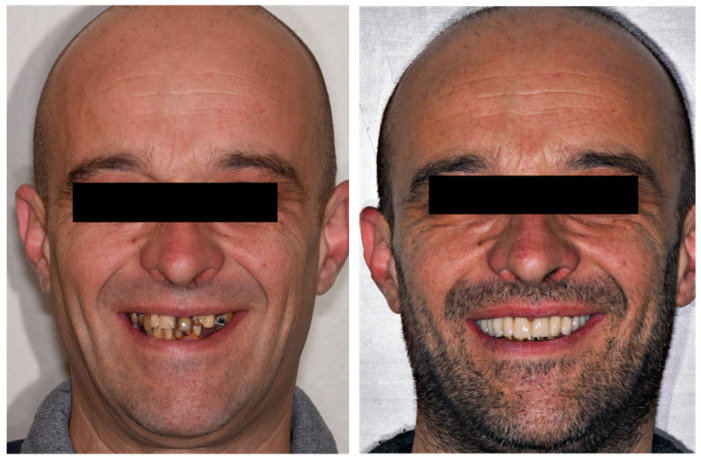
Extraoral pictures taken before treatment (**left**) and at the delivery of the immediate loading prosthesis (**right**) of the same patient.

**Figure 5:**
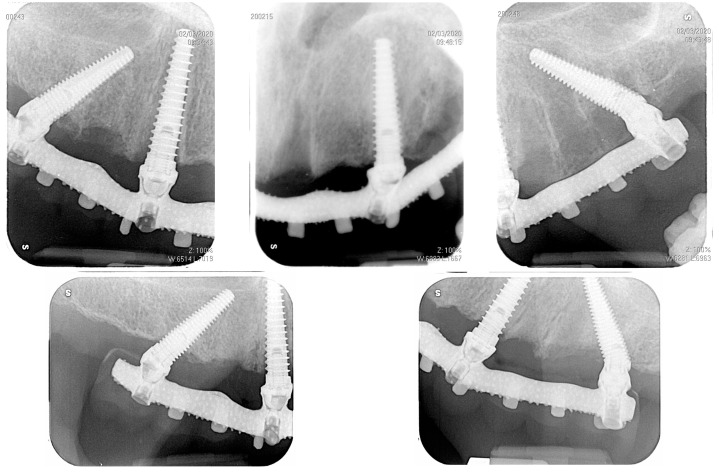
Intraoral radiographs taken at the delivery of the immediate loading prosthesis (T0) and after 24 months in the same patient of Figure 1.

**Figure 6:**
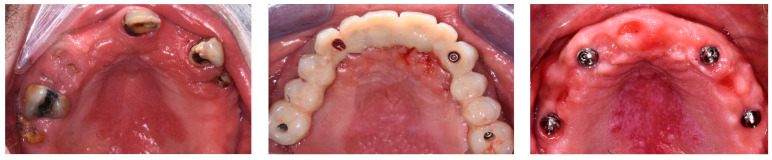
Intraoral pictures taken before treatment (**left**), at the delivery of the immediate loading prosthesis (**center**), and 2 years after surgery (**right**) of the same patient in Figure 2, Figure 3 and Figure 4.

Three patients (two men and one woman) were included in the present research (mean age: 68.7 years; range: 43–86 years) and rehabilitated with full-arch immediate loading rehabilitation of the upper jaw. All the patients were healthy, with no contraindications to implant surgery. Teeth were extracted due to destructive caries in all the three patients. Two of the patients had natural teeth and implant- or tooth-supported fixed partial rehabilitations in the antagonist arch (Figure 1), while the other patient was partially edentulous in the antagonist arch and he was rehabilitated with a removable partial denture in the lower jaw. One of them was a smoker (20 cigarettes/day). A total of 13 implants (3 coaxial and 10 standard) were inserted free hands (Figure 2, Figure 3, Figure 4, Figure 5 and Figure 6, Table 1). One patient had five implants, while the other two patients received four implants each. No patients dropped out at the 24-month follow-up visit.

No implants failed, leading to an implant CSR of 100%. No technical or biological complications occurred during the 24-month follow-up.

Main periodontal indexes are reported in Table 2. No clinically significant differences were identified among the two groups.

The evaluation of the questionnaires filled out by the surgeons showed that no one reported a greater difficulty in positioning the coaxial implants compared to standard implants and they considered the difficulty in implant insertion overlapping (Table 3). Two out of three surgeons reported a greater ease in positioning the conical abutment on the coaxial implant than on the standard one, since a straight abutment was required on coaxial implants and an angulated abutment on standard implants. The surgeons reported that they had no preference for one of the two different implant designs in case of tilted implant insertion. In the free comments area, two out of three surgeons reported that with the coaxial implant, the management of implant depth was influenced by the angulation of the implant shoulder, which dictated the rotation of the implant, and they anecdotally reported that they did not appreciate this aspect.

## 4. Discussion

Full-arch rehabilitations supported by immediately loaded post-extractive implants have proven to be an effective method to restore edentulous or aesthetically and functionally compromised dental arches. However, the procedure of drilling for implant insertion immediately after tooth extraction and subsequent placement of MUAs can present challenges in accurately aligning with the optimal prosthetically guided implant trajectory and are influenced by the existing bone architecture necessary for achieving implant primary stability. Consequently, clinicians may need to explore solutions to enhance implant positioning to achieve parallelism of the supporting implants for optimizing prosthodontic procedures. Numerous potential solutions exist: cement-retained restorations in the anterior region can be a viable approach; however, this may lead to complications like subgingival cement remnants and challenging removal procedures, possibly resulting in peri-implantitis [26,27].

An alternative involves utilizing dynamic screws or the option of employing a transepithelial abutment with adjustable angulation. However, these abutments come with drawbacks: increased laboratory expenses, a more complex prosthodontic rehabilitation process, a relatively weaker connection leading to heightened crown instability, an elevated risk of screw fractures, and a reduction in available prosthodontic space [28]. Lastly, an alternative approach involves using an implant with an inclined shoulder, as proposed in the present study. The inclined shoulder design was introduced in 2007, initially with angulations limited to 12° and 24°, featuring only an external hexagon connection, while nowadays, internal connections are also available, along with greater angulations of the implant shoulder [23,29].

Dental implants with an inclined shoulder macro-design might aid perfect alignment of supporting implants and simplify prosthodontic procedures. Additionally, the implant–abutment connection with standard tilted implants is subcrestal on the distal side, while with the inclined implant shoulder, it is at the bone crest. This might contribute to reducing the risk of peri-implant inflammation and marginal bone resorption.

According to the outcomes of the 3D-FEA by Aktas and Diker [16], the use of coaxial implants also provides biomechanical advantages. Compared to standard implants, the greater bone–implant contact realized using coaxial implants promotes better load distribution at peri-implant bone and implant-prosthodontic components [16]. Although virtual simulation outcomes are challenging to compare with clinical study results due to different study designs, the biomechanical study by Aktas and Diker might offer a potential explanation for optimal clinical outcomes of coaxial implants.

In a recent systematic review, Galve-Huertas [30] analyzed the survival rate and marginal bone loss associated with these implants (12° implant platform). However, only three articles were included with great heterogeneity among them, and the research focused on single implants. The study, involving 60 external hex implants by Southern Implants (such as those used in the present study), revealed a one-year survival rate of 95.9%, accompanied by minimal marginal bone loss, negligible soft tissue recession, and positive papilla index values. However, due to substantial data heterogeneity, a cautious interpretation of the findings was recommended by the authors [30].

In the scientific literature, clinical studies analyzing single [20,23,24,25] or partial implant rehabilitations using implants with an inclined shoulder [28] are available. The outcomes of these works [20,21,23,24,25] indicate that this kind of implant appears to be a viable alternative to standard implants in the rehabilitation of single implants, with a high survival rate and stable bone levels over time.

Results of the present preliminary case report are in accordance with other clinical research on full-arch immediate loading rehabilitation of the upper jaw using tilted implants in terms of implant survival rate and mean bone resorption [8,9,31].

Van Weehaeghe et al. [20] compared conventional tilted implants restored on abutments and implants with an angulated platform restored at the implant level, while in the present study, we chose to restore all the implants at the abutment level. Despite the different prosthodontic approach, Van Weehaeghe et al. [20] reported clinical outcomes similar to the ones herein presented, as no significant differences were found in the clinical parameters evaluated between standard tilted implants and coaxial tilted implants. The possibility to restore coaxial implants directly at the implant level should be considered a further advantage of this implant design.

To the authors’ knowledge, the present study is the first one analyzing coaxial implants with an inclined platform in full-arch immediate loading rehabilitations of the upper jaw. The aim was to evaluate not only the clinical outcomes, but also clinicians’ satisfaction and possible advantages and disadvantages in the use of this implant design. The main limit of the present research is that it is a pilot analysis with a very small sample size since three patients only were involved, providing only a descriptive analysis of the collected data. Both the clinical outcomes and the results of the questionnaire (based on the answers of three different expert clinicians) must be considered preliminary and cannot provide robust scientific evidence. Additionally, considering that full-arch rehabilitations were evaluated, the outcomes of coaxis implant might have been influenced by the other standard implants in the same arch. Randomized controlled clinical trials involving a larger sample of patients and with a power analysis are needed to improve the knowledge and clinical indications for this implant design.

It must also be emphasized that, in addition to the coaxial implants by Southern Implants investigated in the present study, other companies distribute dental implants with an inclined shoulder (i.e., Quatrocone30 by Medentika implant; OsseoSpeed implant by Astra Tech), and the clinical outcomes might not be the same with different implant macro- and micro-designs. In particular, different implant connections are available for both axial and coaxial implants [31]. It must be considered that the external hex connection employed in the present report might help achieve an optimal framework fit also without the use of a conical abutment. However, in the present study, straight or angled conical abutments (MUAs) were used in all the implant sites.

The questionnaire filled out by the clinicians in the present study highlighted a drawback of coaxial implants, that is, a technically more sensitive implant insertion procedure. In fact, during implant placement, the orientation of the inclined shoulder depends on the rotation around the implant axis, which is correlated with the depth of implant insertion. An inaccurate orientation might lead to a more complicated prosthodontic restoration and additional difficulties. As a consequence, while coaxial implants might facilitate the prosthodontic phases, on the other side, they might make implant insertion more challenging, especially in aesthetic areas where optimal implant placement is mandatory for successful outcomes. This underlines the need for an appropriate learning curve for the perfect insertion of coaxial implants, even for surgeons with great experience in the use of standard implants.

## 5. Conclusions

Despite the limitations of the present clinical preliminary report with a very small sample size, external hexagon coaxial implants showed promising clinical outcomes in full-arch immediate loading of the upper jaws of three patients with a 2-year follow-up. This implant design might simplify the prosthodontic procedures; however, a learning curve is needed to optimize their use. Further clinical investigations with a larger sample size and a randomized study design are needed to confirm the present preliminary results.

## Figures and Tables

**Table 1:** Main demographic data.

Patient	Age	Gender	Location	Smoking	Implant Location	Type	Length (mm)	Diameter (mm)	Torque (N/cm^3^)	Tilted/Upright
1	43	M	Maxilla	Yes	16	IBR24D	18	4	50	T
					13	IBT	18	4	40	U
					23	IBT	15	4	40	U
					26	IBT	15	4	40	T
2	86	M	Maxilla	No	16	IBT	18	4	20	T
					13	IBT	13	4	20	U
					11	IBT	15	4	15	U
					23	IBT	18	4	10	U
					26	IBR24D	15	4	30	T
3	77	M	Maxilla	No	16	IBT	18	4	50	T
					13	IBT	18	4	50	U
					23	IBT	15	4	40	U
					26	IBR24D	15	4	40	T

IBR24D: coaxis implant with a 24° inclination; IBT: standard tapered implant.

**Table 2:** Mean (SD) values for the parameters of peri-implant health recorded for coaxial and standard implants. m = months, MBL = mean bone loss, PPD = probing pocket depth, BOP = bleeding on probing, PI = plaque index.

Time	Coaxial	Standard
MBL 3 m (mm)	0.4	0.3
MBL 6 m (mm)	0.7	0.6
MBL 12 m (mm)	0.9	0.7
MBL 24 m (mm)	1.1	1.0
PD 3 m (mm)	1.83	1.25
PD 6 m (mm)	2	1.33
PD 12 m (mm)	2.08	1.5
PD 24 m (mm)	2.08	1.75
BOP 3 m	1.7	0.7
BOP 6 m	0.7	0.3
BOP 12 m	1	0.4
BOP 24 m	1	1.4
PI 3 m	1	1
PI 6 m	1	0.92
PI 12 m	1	0.8
PI 24 m	1	0.94

**Table 3:** Questionnaire results.

	Operator	Operator 1	Operator 2	Operator 3
1	Did you encounter any difficulties during the insertion of the standard implant?	no	no	no
2	Did you encounter any difficulties during the insertion of the coaxial implant?	no	no	no
3	Did you encounter more difficulty during the insertion of the standard or of the coaxial implant?	overlapping	overlapping	overlapping
4	With which implant design did you have more difficulty during the multiunit abutment connection?	traditional	traditional	overlapping
5	Do you think that the coaxial implant facilitated the optimization of the prosthodontic axes?	no	no	no
6	During future interventions requiring the insertion of tilted implants, which implant type would you prefer?	overlapping	overlapping	overlapping

## Data Availability

The raw data supporting the conclusions of this article will be made available by the authors on request.
